# Randomized Clinical Trial of a Self-Adhering Flowable Composite for Class I Restorations: 2-Year Results

**DOI:** 10.1155/2017/5041529

**Published:** 2017-03-01

**Authors:** J. Sabbagh, S. Dagher, N. El Osta, P. Souhaid

**Affiliations:** ^1^Department of Restorative Dentistry, Lebanese University, Beirut, Lebanon; ^2^Department of Pedodontics, Saint-Joseph University, Beirut, Lebanon; ^3^Department of Public Health, Saint-Joseph University, Beirut, Lebanon; ^4^Dental College, Beirut, Lebanon

## Abstract

*Objectives.* To compare the clinical performances of a self-adhering resin composite and a conventional flowable composite with a self-etch bonding system on permanent molars. The influence of using rubber dam versus cotton roll isolation was also investigated.* Materials and Methods.* Patients aged between 6 and 12 years and presenting at least two permanent molars in need of small class I restorations were selected. Thirty-four pairs of restorations were randomly placed by the same operator. Fifteen patients were treated under rubber dam and nineteen using cotton rolls isolation and saliva ejector. They were evaluated according to the modified USPHS criteria at baseline, 6 months, and 1 and 2 years by two independent evaluators.* Results.* All patients attended the two-year recall. For all measured variables, there was no significant difference between rubber dam and cotton after 2 years of restoration with Premise Flowable or Vertise Flow (*p* value > 0.05). The percentage of restorations scored alpha decreased significantly over time with Premise Flowable and Vertise Flow for marginal adaptation and surface texture as well as marginal discoloration while it did not vary significantly for color matching. After 2 years, Vertise Flow showed a similar behaviour to the Premise Flowable used with a self-adhesive resin system.

## 1. Introduction

It has always been one of the major aims of paediatric dentistry to prevent caries in the young population. Upon eruption, molars anatomy shows grooves and pits favourable for caries development. The use of fissure sealant and preventive resin restoration (PRR) is a very common practice aiming to reduce caries incidence.

The important campaigns and educational measures taken by health authorities to decrease caries incidence did not decrease the use of fissure sealants. It still indicated for young patients, as a preventive measure to protect their teeth. White resin sealant was widely used which allows its detection. Despite the precautions taken during placement (isolation, etching, rinsing, and so forth) sealant application has demonstrated in long-term clinical evaluation many shortcomings such as discolorations, secondary caries, and loss of the restoration [1, 2]. Gibson et al. using a split half-mouth design showed that fissure sealants caused a 51% reduction in caries, despite only two-thirds remaining intact [3]. Placing fissure sealants successfully is highly dependent upon operator skill and patient cooperation [4]. A long-term retrospective study showed that, after 11.6 years, only 41.3% of placed sealants were fully retained. Differences in the long-term performance of sealants depended on tooth type, (premolars versus molars) and restorations profile of the individual patient [5]. As the sealant replaces “extension for prevention,” it requires regular monitoring and maintenance [2].

Preventive resin restoration or PRR is a technique widely used to fill the grooves of permanent molars upon or after eruption and remove any initial caries. These restorations are indicated when occlusal caries have involved a minimal amount of dental tissues.

Since their introduction in 1996, flowable composites are widely used for sealants and PRR restorations. They are available in different shades, allowing better aesthetics, and contain a higher percentage of fillers than fissure sealant and as a result have better mechanical properties [6].

Patients' age and cooperation are not always ideal; the treatment outcome is greatly influenced by the patients' behaviour. It is therefore important to reduce the application time of some materials mostly in paediatric dentistry. Resin based materials and technologies have widely evolved in that direction facilitating the placement of composite restorations.

Depending on the type of adhesive system used, application duration may take from 36 to 115 seconds. The number of steps also varies between 5 and 12, according to manufacturer's instructions [7].

Self-etch adhesives when compared to total-etch systems have the advantage of reducing the application time and the number of steps. Few years ago, self-adhering flowable composites were introduced to the market to reduce procedure steps. Two products are now available on the market: Vertise Flow (Kerr, Orange, USA) and Fusio Dentine (Generic Pentron, USA). Vertise™ Flow composite is available in nine shades, is radiopaque, and has numerous indications including small class I restorations; base/liner for class I and II restorations; and, in paediatric dentistry, pit-and-fissure sealant or preventive resin restoration. Although many in vitro investigations were conducted on Vertise Flow [8–11] only two studies have evaluated their clinical behaviour at 6 months [12] and two years [13].

The aim of this study was to compare the clinical behaviours at two years, of a self-adhering composite to a conventional flowable composite used with a self-etch adhesive system and assess the effect of field isolation using rubber dam versus cotton rolls.

## 2. Methods and Materials

**Figure 1:**
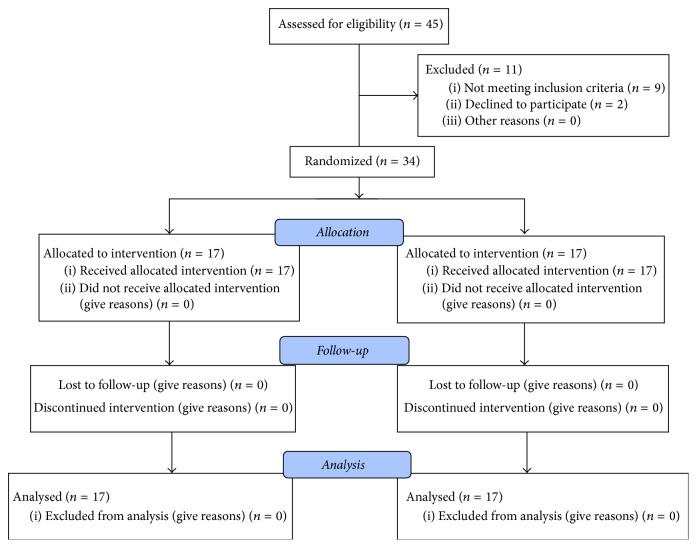
Consort flow diagram showing the process of case selection.

Patients aged between 6 and 12 years and presenting with at least two permanent molars in need of small class I restorations (average 1,5 mm) were selected for this study and treated in a paediatric clinic. The consort flow diagram in Figure 1 shows the number of individuals enrolled, treated, and evaluated during this study. The exclusion criteria included deep carious defects, disabled patients, or those presenting a compromised medical history, lack of compliance, or allergic history to methacrylates. All procedures performed in this study, involving human participants, were in accordance with the ethical standards of the institutional and/or national research committee and with the 1964 Helsinki Declaration and its later amendments or comparable ethical standards. Informed consent was obtained from all patients' parents study. Then, each pair of teeth was treated randomly according to two different techniques.

All cavities and restorations were prepared and placed by the same experienced operator. Small class I restorations, less than 2 mm depth, were prepared with a high speed with copious irrigation without any bevel and did not involve any functional areas. Round diamond burs (SS White, USA) with a diameter of 1 mm and 3 mm length were used.

One tooth was restored using the self-adhering composite, Vertise Flow (Kerr, Orange USA), and the other one using Premise Flowable with OptiBond All-In-One self-etch bonding system (Kerr, Orange, USA). A Demi LED (Light Emitting Diode) light-curing unit, monitored regularly using a Demetron LED radiometer (Kerr, Orange, USA) with a minimum intensity of 800 mW/cm^2^, was used for the polymerization during 20 seconds. The composition of the materials and the application procedures are shown in Table 1.

After removing the rubber dam, the occlusion was checked with articulating paper. Finishing and polishing of the restorations were performed immediately using water-cooled fine diamond burs followed by Optibrush polishing points (Kerr, Bioggio, Switzerland) on a slow speed contra angle without irrigation. In total, thirty-four pairs of restorations (*n* = 68) were randomly placed. Fifteen patients were treated under rubber dam and nineteen using cotton rolls isolation and saliva ejectors.

### 2.1. Clinical Evaluation

The restorations were blindly evaluated according to the modified criteria of United Stated Public Health Services (USPHS criteria) shown in Table 2. The evaluations were conducted at baseline (one week after placement), 6 months, and 1 and 2 years by two independent evaluators. Prior to each evaluation session, an interevaluator calibration was performed. When a difference was observed between the evaluators, a second evaluation was undertaken, and the results were discussed till a forced consensus was reached.

### 2.2. Statistical Analysis

The statistical analysis was performed using a software program (SPSS for windows version 18.0, Chicago, IL, USA). The alpha error was set at 0.05. Measured variables of the study were marginal adaptation, surface texture, anatomical form, marginal discoloration, and color match. For statistical analysis, Fisher Exact Tests were used to explore significant difference within time between groups (1 week, 6 months, 1 year, and two years).

## 3. Results

All patients attended the two-year recall and all the restorations were evaluated. At the 2-year recall, three Vertise Flow and one Premise Flowable restorations were lost and one Premise Flowable composite was broken. For all measured variables, there was no significant difference between rubber dam and cotton after 2 years of restoration with Premise Flowable (*p* value > 0.05) or Vertise Flow (*p* value > 0.05).

**Figure 2:**
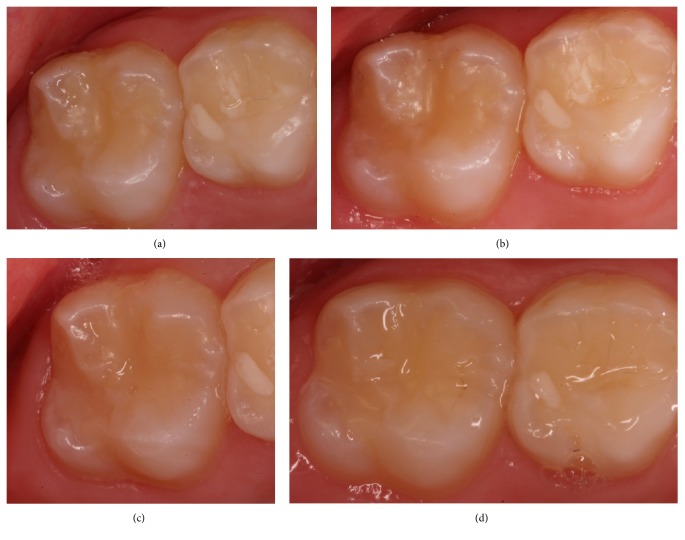
It shows an Alpha rated case of Vertise Flow at different evaluation times ((a) = 1 week, (b) = 6 months, (c) = 1 year, and (d) = 2 years).

**Figure 3:**
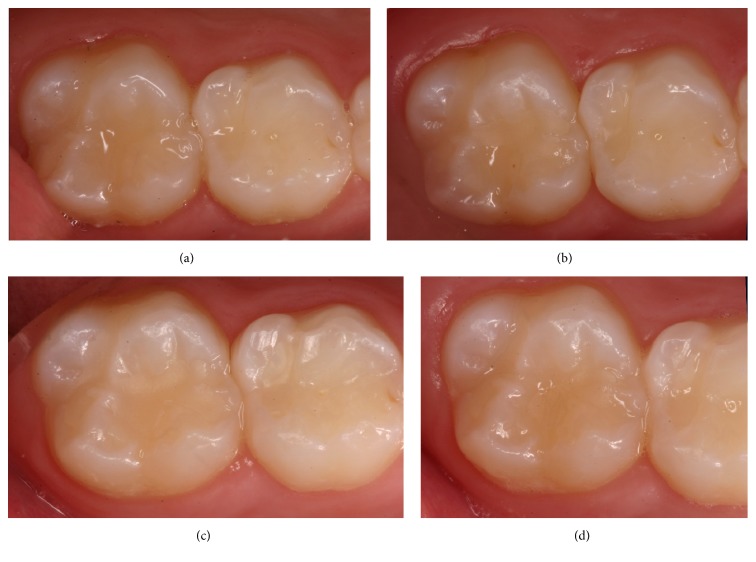
It shows an Alpha rated case of Premise Flowable at different evaluation times ((a) = 1 week, (b) = 6 months, (c) = 1 year, and (d) = 2 years).

The percentage of restorations scored alpha decreased significantly over time with Premise Flowable (*p* value < 0.0001) and Vertise Flow (*p* value < 0.0001) in terms of marginal adaptation, surface texture, and marginal discoloration. However, no significant difference was found between Vertise Flow and Premise Flowable (*p* value > 0.05). Concerning the anatomical form, the percentage of restorations scored alpha decreased significantly over time with Vertise Flow (*p* value = 0.002). This was not the case with Premise Flowable (*p* value = 0.066). There was no significant difference in color over time with Premise Flowable (*p* value = 0.112) and Vertise Flow (*p* value = 0.392). Figures 2 and 3 show two alpha rated cases at the different evaluation periods, using the two investigated composite systems.

A summary of Alfa rated restorations according to the type of resin composite is shown in Table 3.

## 4. Discussion

The use of a self-adhering composite is an innovative step in restorative dentistry for young patients. To date, few studies have been published concerning this new category of materials and most of the studies concern the use of sealants. The drawback of those studies is that the success rates are not easily comparable from study to study as the definition of failure was reported as actual presence of caries or loss of sealant.

As a product is developed, a wide variety of properties are measured in the laboratory in order to evaluate its performance and to try to predict its “clinical longevity.” Materials are evaluated through two kinds of tests, in vitro tests and in vivo tests, each having its advantages and disadvantages.

In vitro studies are undertaken in laboratories where conditions are quite different from clinical situations. The link with long-term clinical behaviour is complex and difficult to predict and the different fatigue factors (thermal, chemical, and mechanical) that occur in the oral environment are not taken into consideration [14, 15].

In vivo evaluation or clinical studies are more interesting and relevant. They reflect the clinical behaviour and performance of the material in the oral cavity under variable conditions: biological (saliva, dental plaque), chemical (pH), physical (temperature), and mechanical stimulations. During the evaluation procedure, the Alpha and Bravo rating are considered acceptable while Charlie and Bravo are considered unacceptable.

In order to overcome the interoperator variability, the two materials were randomly applied to every patient and the evaluation process was blind, minimizing the risk of bias.

Vertise™ Flow is the first self-adhering resin composite from Kerr, which includes in its formulation the OptiBond technology. It is the logical continuum in the chain of product development aiming towards simplification and ease of application. The bonding mechanism with tooth structure is a chemical bonding achieved via the GPDM (glycerophosphate dimethacrylate) between phosphate functional groups of GPDM monomers and calcium ions of enamel and dentine [16].

By including the bonding in its formulation, self-adhering resin composites eliminate the additional steps of etching/priming/bonding, otherwise necessary to bond a resin composite to dentin and enamel. In the treatment of young patients, mostly those showing a difficult behaviour, the use of this category of materials may be very useful.

This product has been evaluated using in vitro and in vivo testing for approximately two years. The first clinical study was conducted on 40 class I restorations at the university of Siena, Italy [12]. At the 6-month recall, the 40 restorations were reevaluated. Out of the 40 restorations using Vertise™ Flow self-adhering material, only two restorations showed Bravo score and 1 Charlie score for marginal discoloration and integrity. All other parameters showed Alfa scores. No postoperative sensitivity was recorded at any of the recalls. Recently a 2-year clinical trial evaluated the retention rates of two different pit-and-fissure sealants compared with a flowable composite and a self-adhesive flowable composite over a 24-month period. The flowable composite used with an adhesive system was found to be superior to other sealing materials with a retention rate of 95.7 compared to 62.2% for Vertise Flow.

Including an adhesive component in the formulation of self-adhering composites may have some adverse effects on the physical behaviour of the composite. A recent in vitro study has compared the hygroscopic absorption characteristics of different resin based materials. The self-adhering composite was the least dimensionally stable, due to the incorporation of hydrophilic monomers [8].

According to the manufacturer, the percentage of fillers by weight of Vertise Flow is 70% and 72.5% for Premise Flowable (Table 1). Thus, the mechanical behaviour of the two products is expected to be comparable.

Achieving a successful and durable bonding procedure involves many steps and is technique-sensitive and time-consuming. Usually, dental manufacturers include in every adhesive kit a leaflet explaining the instructions for use. Most of the time, dentists do not read it and consequently do not follow precociously the steps, or application time. Deviation from these procedures leads to incomplete resin penetration and may result in both reduced bond strength and micro- or nanoleakage [17]. Clinically this may cause postoperative sensitivity after composite placement.

Another parameter of paramount importance during the restorative phase is the field of isolation. The use of rubber dam, although highly recommended, is seldom used by dentists (10%). The external humidity may affect adversely the bonding process if the isolation is not adequate.

Pedodontists often have other clinical difficulties including behaviour problems of their young patients. Their requirements for a material include the possibility of applying a material in a minimum amount of time and if possible shorten the duration of the procedure.

Many studies have been conducted regarding the stability of self-etch systems [18]. Their shelf life is shorter compared to the total-etch systems, and their stability is questionable, as the different components may undergo phase separation and show the formation of droplets [19]. Furthermore, solvent evaporation is a major problem encountered with adhesive systems. Depending on the type of solvent, water, alcohol, or acetone based adhesives systems, they often show variable sensitivity to temperature and to evaporation. Compared to orthophosphoric acid at 37% (pH = 0.1), Vertise Flow is considered mild and less aggressive with a pH of 1.9. This explains the fact that unprepared enamel needs to be roughened with a diamond bur or etched during 20 sec with phosphoric acid (37%) prior to the application of Vertise Flow, to enhance the bonding mechanism on enamel rods. A recent study conducted by Eliades et al. [20] confirmed those finding and concluded that the low flow of self-adhesive composites affected their fissure penetration capacity. When combined with enamel acid-etching, adaptation and microleakage scoring were substantially improved in comparison with enamel air particle abrasion. This concept is called selective etching and may be extended to the use of Vertise Flow.

In the present clinical study, the use of rubber dam did not influence significantly the results. No etching was applied on the cavities as the enamel was prepared with a diamond bur. The application procedure was followed precisely according to the manufacturer's recommendations and the product kept refrigerated.

The two resin based materials investigated in the present study showed similar retention rates. Previous studies compared the retention rates of sealants to flowable composites [21–23].

Except one study that showed relatively low retention rates (50 to 75%) in deciduous molars of 7-year-old children, after one year [21], the retention rates were above 95% which is in accordance with the findings of the present study. However, it was shown that the use of bonding agents improved the retention rate of sealant restorations [24].

Vertise Flow has been under in vitro and in vivo investigations for almost four years. The first clinical study conducted on class I restorations showed satisfactory results [13]. Out of the 40 restorations made with Vertise Flow, only two showed Bravo scores and one Charlie score for marginal discoloration and integrity. All other parameters showed Alfa scores. No postoperative sensitivity was recorded at any of the recalls. The second clinical study was less favourable towards self-adhesive resin composites and at 2 years showed better retention rate for the conventional flowable composite applied with an adhesive system. More long-term clinical studies are needed to validate those finds.

## 5. Conclusion

- At 2 years, the Vertise Flow showed a similar behaviour to the Premise Flowable used with a self-etch adhesive system (OptiBond All-In-One).
- The use of rubber dam did not affect the clinical behaviour of the materials.

## Figures and Tables

**Table 1:** Composition and application procedure of the materials used.

Material	Composition	Application
Vertise Flow (Kerr, USA) Lot # 1000010715	*Matrix*: GPDM adhesive monomer, UDMA, BisGMA, and other methacrylate comonomers, photoinitiators *Fillers*: 70% by weight. Ytterbium Fluoride, barium aluminosilicate glass, prepolymerized fillers, and colloidal silica	(1) Brush the layer with moderate pressure for 15–20 seconds to obtain a thin layer (<0,5 mm) (2) Light-cure for 20 seconds (3) Place additional increments of Vertise Flow in 2 mm or less (4) Light-cure for 20 seconds

Premise Flowable (Kerr, USA) Lot # 1000010505	*Matrix*: Bis-GMA, ethoxylated bis-phenol-A-dimethacrylate, TEGMA, light-cure initiators, and stabilizers *Fillers*: 72,5% by weight. Barium glass, prepolymerized fillers, and silica nanoparticles	(1) Apply in increment of 2 mm or less (2) Light-cure for 10 seconds

OptiBond All-In-One (Kerr, USA) Lot # 3354615	Glycerol phosphate dimethacrylate (GPDM), mono- and difunctional methacrylate monomers, water, acetone, ethanol, nanofillers, and camphorquinone (CQ)	(1) Apply one coat and scrub it using a disposable microbrush for 20 seconds (2) Repeat step (1) (3) Dry with air for 5 seconds (4) Light-cure for 10 seconds

**Table 2:** Modified criteria of United Stated Public Health Services (USPHS criteria) used to evaluate the restorations.

Category	Rating and criteria
Marginal adaptation	A: explorer does not catch B: explorer catches, no crevice is visible into which explorer will penetrate C: obvious crevice at margin, enamel, dentin, or base exposed D: restoration mobile, fractured, or missing

Surface texture	A: no surface porosities or cracks B: slight surface porosities or cracks C: obvious surface porosities or cracks

Anatomical form	A: the restoration is continuous with tooth anatomy B: Slightly under- or overcontoured restoration; marginal ridges slightly undercontoured; occlusal height reduced locally C: wear beyond the DEJ (clinically unacceptable) D: restoration is missing partially or totally; fracture of tooth structure; showing traumatic occlusion

Marginal discoloration	A: no discoloration anywhere along the margin B: superficial staining (removable, usually localized) C: deep staining (not removable, generalized)

Color match (immediately after placing the restoration)	A: no shade mismatch in room light in 3-4 seconds B: perceptible mismatch but clinically acceptable C: obvious mismatch, esthetically unacceptable

**Table 3:** Number and percentage of restorations that scored Alfa at baseline (BL), six months, and one year and two years for each parameter.

	Premise (*n* = 34)				Vertise (*n* = 34)			
	BL	6 months	1 year	2 years	BL	6 months	1 year	2 years
Marginal adaptation	30 (88.2%)	25 (73.5%)	22 (64.7%)	19 (55.9%)	27 (79.4%)	22 (64.7%)	20 (58.8%)	17 (50.0%)
Surface texture	31 (91.2%)	29 (85.3%)	29 (85.3%)	25 (73.5%)	32 (94.1%)	32 (94.1%)	29 (85.3%)	26 (76.5%)
Anatomical form	32 (94.1%)	32 (94.1%)	31 (91.2%)	29 (85.3%)	33 (97.1%)	32 (94.1%)	32 (94.1%)	27 (79.4%)
Marginal discoloration	33 (97.1%)	33 (97.1%)	33 (97.1%)	28 (82.4%)	34 (100%)	32 (94.1%)	31 (91.2%)	28 (82.4%)
Color match	34 (100%)	32 (94.1%)	32 (94.1%)	31 (91.2%)	34 (100%)	34 (100%)	33 (97.1%)	33 (97.1%)
